# Novel mutation in the *NDP* gene associated with Norrie disease in a Chinese pedigree

**DOI:** 10.1002/mgg3.2345

**Published:** 2023-12-26

**Authors:** Keke Jiang, Shuying Wang, Huixin Sun, Chuanzhi Peng, Nan Li, Shuchan Li, Rongyu Gao, Jie Zhang

**Affiliations:** ^1^ Department of Ophthalmology, Zhengda Guangming Ophthalmology Group Weifang Eye Hospital Weifang China; ^2^ Department of Ophthalmology Gaomi People's Hospital Weifang China; ^3^ Department of Ophthalmology Weifang Medical University Weifang China

**Keywords:** c.174G>C, ND, *NDP* gene, pedigree, sequencing

## Abstract

**Purpose:**

Norrie disease (ND) is a rare X‐linked recessive disorder characteristic of early childhood blindness. While several mutations in the *NDP* gene have been reported as causative for ND, the genetic etiology remains unknown for many patients. This study aims to describe a novel mutation and explore the clinical manifestations in a Chinese family with two affected males.

**Methods:**

Exome sequencing (ES) was employed to identify the causative gene in a four‐generation pedigree. Sanger sequencing was subsequently utilized to validate the mutation detected by ES in additional family members. Ophthalmologic examination and diagnostic imaging relevant to ND were conducted.

**Results:**

The proband (IV:2), an 8‐month‐old male infant, presented with binocular retinal detachment. DNA sequencing revealed a novel heterozygous missense mutation (c.174G>C) within the *NDP* gene in the proband. This mutation affected highly conserved residues and was predicted to disrupt the normal protein structure. Furthermore, the variant co‐segregated with the disease phenotypes within the family.

**Conclusions:**

Our findings identified a novel missense mutation in the NDP gene associated with Norrie disease in China, expanding the mutation spectrum associated with ND. This discovery holds diagnostic, prognostic, and genetic counseling implications for affected individuals.

## 1 INTRODUCTION

Norrie disease (ND; OMIM 310600) is a rare X‐linked genetic disorder first reported in1927. It is characterized by congenital blindness and white pupils (Krill, 1967; Norrie, 1927), with some infants exhibiting a yellow‐white massive microstructure with well‐developed vasculature in the posterior vitreous at birth or soon after. As the disorder progresses, the eyeballs may shrink, the lens becomes cloudy, and cataracts eventually form (Chen et al., 1992). The exact, annual incidence and prevalence of ND are unknown, but more than 400 cases have been detailed in the literature, and approximately 30%–50% of patients experience varying degrees of neurodevelopmental retardation with cognitive impairment and/or behavioral disorders (Huang et al., 2017). Additionally, one‐third of patients develop progressive sensorineural deafness due to vascular and cochlear abnormalities (Riveiro‐Alvarez et al., 2005; Rodríguez‐Muñoz et al., 2018). The disease does not show any specific racial or ethnic associations, although affected individuals are predominantly male, while females typically serve as carriers (Huang et al., 2017).

The Norrie disease protein (*NDP*) gene (OMIM 300658) often result in a severe phenotype is located on chromosome Xp11.3 (ChrX: 4,36,92,969–4,37,17,694) (GRCh37) and is considered the pathogenic basis of ND (Berger et al., 1992; Chen et al., 1992). The Norrie protein, a secreted protein with a cystine‐knot motif, mediates the activation of the Wnt/β‐catenin pathway and regulates cell division and differentiation. It is widely expressed in various tissues, such as the eye, ear, and brain and plays a crucial role in the development of the retina, cochlea, and central nervous system (Warden et al., 2007; Xu et al., 2004). The mouse models demonstrate that in the absence of norrin, radial extension of the superficial retinal vasculature is significantly slowed and the vessels have a porous blood‐retinal barrier. In addition, the intermediate and deep retinal capillary layers fail to develop (Schäfer et al., 2009).

As there are no biochemical or functional tests currently used as disease markers, the diagnosis of ND is based on a combination of clinical ocular manifestations and molecular genetic testing. Despite the identification of various *NDP* defects, including missense, nonsense, frameshift, or deletions, there remain unexplained cases. Therefore, the identification of new mutations is essential for the clinical and genetic diagnosis of ND.

Exome sequencing (ES) is a targeted gene panel that covers most of the genes in the Online Mendelian Inheritance in Man (OMIM) database (Amberger et al., 2015). ES is primarily used to identify and study variations in coding regions and UTR regions associated with disease and population evolution. By combining exon data from numerous public databases, it becomes advantageous to elucidate the relationship between variation and disease. In this study, we present a family with prominent symptoms suspected of having ND. ES and Sanger sequencing were employed to perform genetic analysis in the family pedigree, and a novel *NDP* mutation (c.174G>C) was identified though these methods. Subsequent bioinformatics analyses predicted this mutation to be a pathogenic variant.

## 2 MATERIALS AND METHODS

### 2.1 Patients, editorial policies, and ethical considerations

**FIGURE 1:**
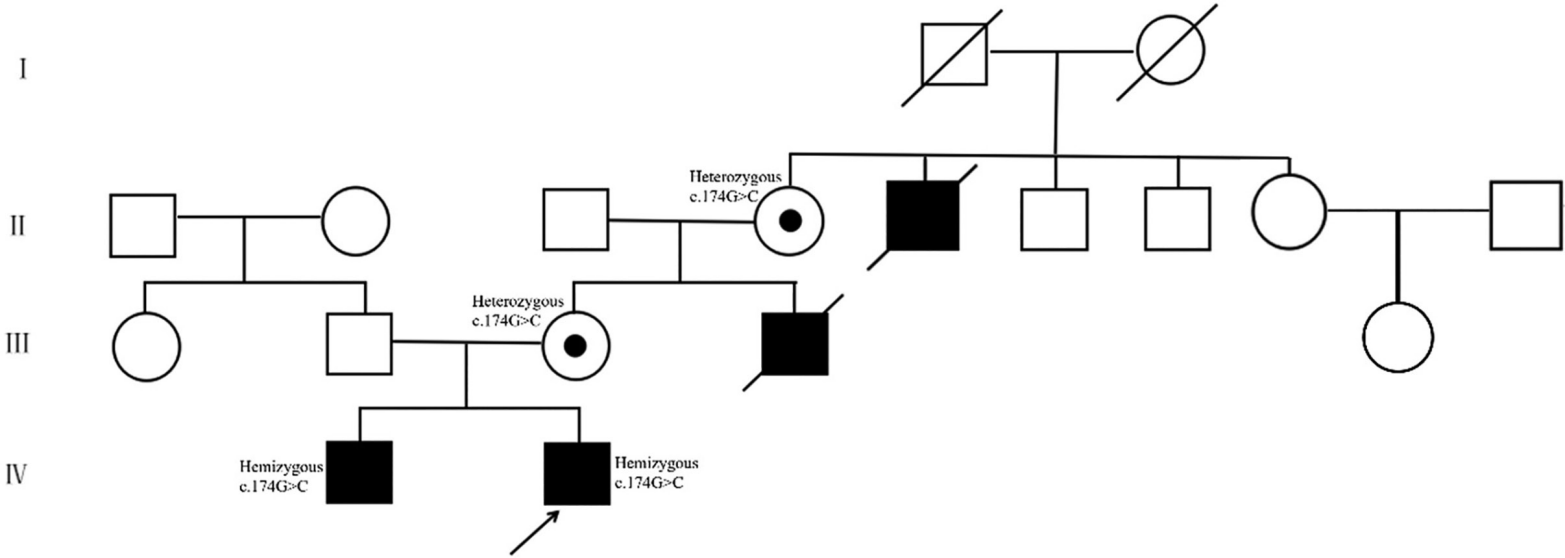
Pedigrees of the families with mutations. Squares indicate men and circles women; black and white symbols represent affected and unaffected individuals, respectively. The proband is marked with an arrow.

**FIGURE 2:**
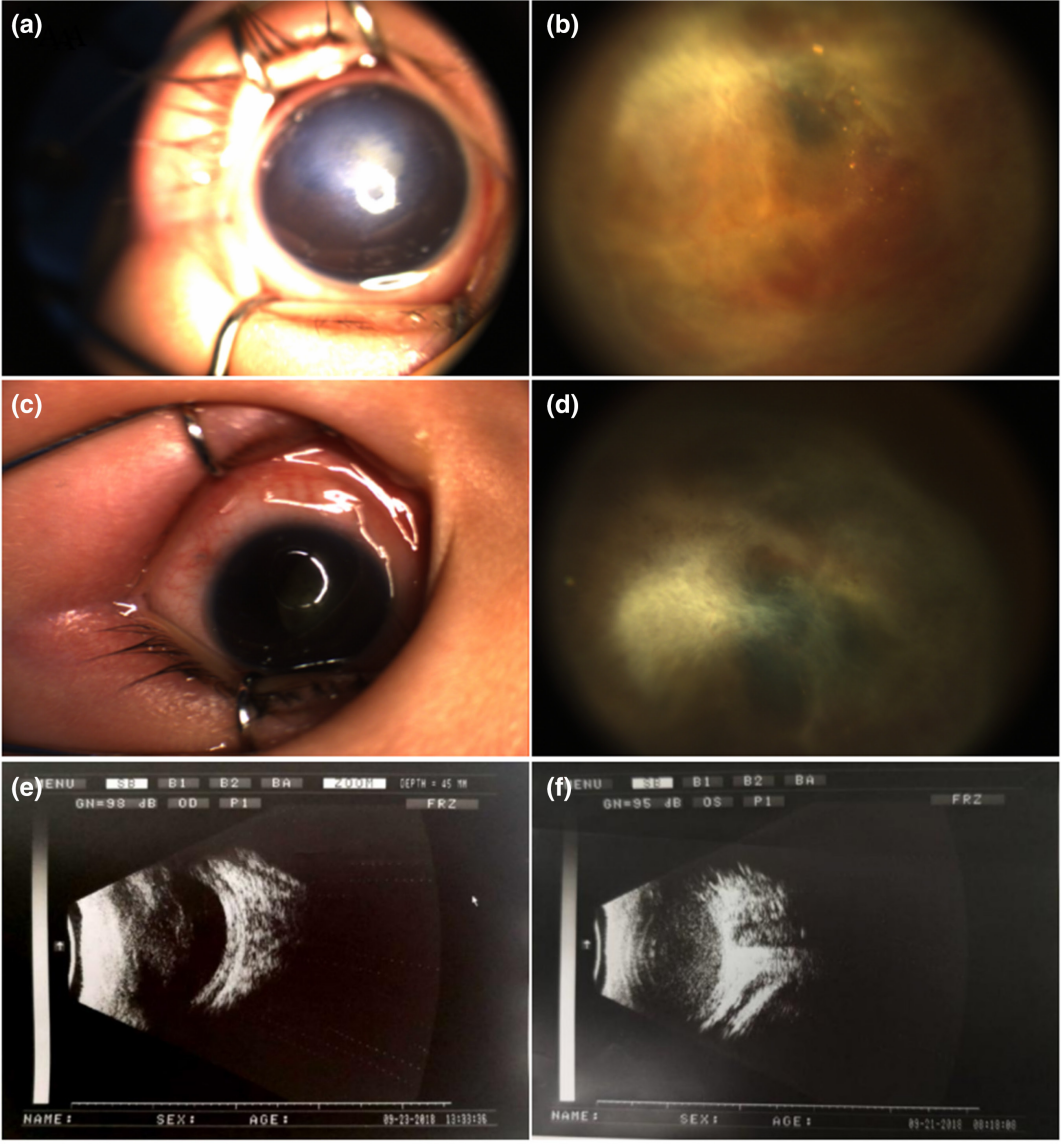
Photographs of the anterior segment and B‐mode ultrasonography findings of case IV‐2: (a, b, e) represent the right eye, (c, d, f) represent the left eye. (a, b) The photographs of right eye showed no light sensation, a white spot about 3 × 3 mm, the anterior chamber disappeared, the pupil was not round, part of the iris was attached to the inner skin of the cornea, the lens was mildly cloudy, a large amount of golden granules were hidden in the vitreous cavity, and retinal detachment was hidden in the fundus. (c, d) The left eye showed no light sensation, the cornea was clear, the anterior chamber was deep, the pupil was not round, the crystal was cloudy, retinal hemorrhage was hidden in the fundus, and total retinal detachment was observed. (e) The morphology of right eyeballs was fine, and diffuse spot cluster echo was visible in the vitreous cavity. (f) Diffuse mass echoes are seen in the vitreous cavity of the left eye.

For this study, we recruited a four‐generations family from Shandong Province, China, comprising 17 individuals, including two affected males (Figure 1). The proband, an 8‐month‐old male infant, displayed the classical features of ND, such as white pupils and binocular retinal detachment (Figure 2). Complete clinical ophthalmic examination was conducted on all patients. Written informed consent was obtained from all participants, who were also aware of their involvement in the publication of the study. The study protocols involving human subjects were approved by institutional ethics committees of Weifang Eye Hospital and adhered to the principles outlined in the Declaration of Helsinki.

### 2.2 Mutation screening by next generation sequencing

Genomic DNA samples from affected patients and healthy controls were extracted from peripheral blood using the QIAamp DNA Blood Mini Kit (Qiagen, Hilden, Germany) following the standard manufacturer's instructions. Library preparation and next‐generation sequencing was performed at the MyGenostics, Beijing, China. The quality and enrichment of the resulting products were assessed using an Agilent 2100 Bioanalyzer. Subsequently, all amplified libraries were sequenced on the MGIseq‐2000 platform. The sequencing reads were automatically demultiplexed using the index. Mutation analysis of the *NDP* gene was conducted at MyGenostics, Beijing, China.

### 2.3 Bioinformatics analysis and molecular evolution analysis

Sequencing data were aligned to UCSC hg19 human reference sequence using the Burrows‐Wheeler aligner (BWA‐MEM, version 0.7.10) (Li & Durbin, 2010). Variants calling was performed using Genome Analysis Tool Kit (GATK, version 3.3) (McKenna et al., 2010). All identified variants were annotated using Annovar (Wang et al., 2010) and SnpEff (Cingolani et al., 2012). Subsequently, the variants obtained from the above pipeline were subjected to additional filtered to exclude benign variants with minor allele frequency (MAF) >0.1% in databases such as 1000 Genomes (Siva, 2008), dbSNP (Sherry et al., 2001), EXAC (Lek et al., 2016), ESP6500database (Tennessen et al., 2012), and internal data (MyGenostics). Finally, variant prioritization and selection were performed combining total depth, quality score, MAF, potential deleterious effect, and the existence of mutation reports in common databases such as the Human Gene Mutation Database (Stenson et al., 2009), the Retinal Information Network (Daiger et al., n.d.), ClinVar (Landrum et al., 2014), or the Online Mendelian Inheritance in Man. These criteria were used to evaluate the confidence of the variant calling. Finally, a disease‐associated mutation in the *NDP* gene was identified following the guidelines set forth by the American College of Medical Genetics (ACMG) protocol (Richards et al., 2015).

To evaluate the deleteriousness of the novel variation c.174G > C in the *NDP* gene, several online tools (SIFT, Mutation Taster, Polyphen2 HDIV, Polyphen2 HVAR, Mutation Assessor) were employed (Table I). Furthermore, the SWISS‐MODEL workspace was utilized to predict the effect of the variant on the protein structure (Wawrzynski et al., 2022). Additionally, a phylogenetic approach was employed to assess the evolutionary conservation of the candidate mutation (Figure 4).

### 2.4 Sanger sequencing

To validate and detect the novel variant in the proband (IV:2) and other family members, conventional Sanger sequencing was conducted. Polymerase chain reaction (PCR) was performed to amplify the target region, and the resulting PCR product was subjected to direct sequencing on a 3500XL Genetic Analyzer (Applied Biosystems, Foster City). The primers sequences used for PCR were designed using Primer Premier 5.0. The forward primer sequence was (5′‐GGAGGTGAAGCCATTTCCAATT‐3′) and the primer sequence was (5′‐CTTGCCTGTTTCTGAGGG‐3′).

## 3 RESULTS

### 3.1 Clinical manifestations

In this study, the proband was referred to our hospital for treatment after being diagnosed with retinal detachment in both eyes due to “white eyeball” at a local hospital 2 months after birth. The child, born at full term of normal weight, was the second child of the mother, who denied any history of drugs, alcohol consumption, or illness during pregnancy. The mother had an uneventful pregnancy, and the parents were not closely related.

Upon clinical examination, the proband exhibited stable vital signs, normal growth and development, and an inability to evaluate intellectual development. Binocular visual acuity examination revealed no perception of light, and the intraocular pressure, measured by Icare Pro, was 17.3 mmHg (right eye) and 16.5 mmHg (left eye). RetCamIII examination showed the following finding: a 3 × 3 mm whited spot in the lower temporal cornea of the right eye, mild corneal edema, absence of the anterior chamber, irregularly shaped pupils, partial adhesion of the iris to the inner surface of the cornea, mild lens opacity, presence of abundant golden granules in the vitreous cavity, and hidden retinal detachment in the fundus (Figure 2a,b). In the left eye, the cornea was clear, the anterior chamber was deep (Figure 2c), the pupil was irregularly shaped, the lens was opaque, retinal hemorrhage was observed in the fundus, and total retinal detachment was evident (Figure 2d). Ophthalmic B‐ultrasound examination revealed well‐defined morphology of both eyeballs, with the right eye showing diffuse spot cluster echoes in the vitreous cavity, exhibiting movement (+) and post‐movement (+) (Figure 2e). The left eye displayed diffuse mass echoes in the vitreous cavity (Figure 2f).

Furthermore, it was noted that the child's uncle and great‐uncle had been blind since childhood, with specific details of their conditions remaining unknown. Both individuals unfortunately passed away at approximately 10 years old. Additionally, the child's brother had also been blind since childhood. Eye examination of the parents showed no abnormality. A thorough examination of the family history revealed the presence of other affected individuals, all of whom were male. Based on the combination of the clinical phenotypes observed in the retina and genetic pattern observed within the family, a strong suspicion of Norrie disease (ND) arose. Consequently, the child's parents were advised to undergo genetic testing, and all family members willingly participated and provided informed consent.

### 3.2 Mutation analysis

**FIGURE 3:**
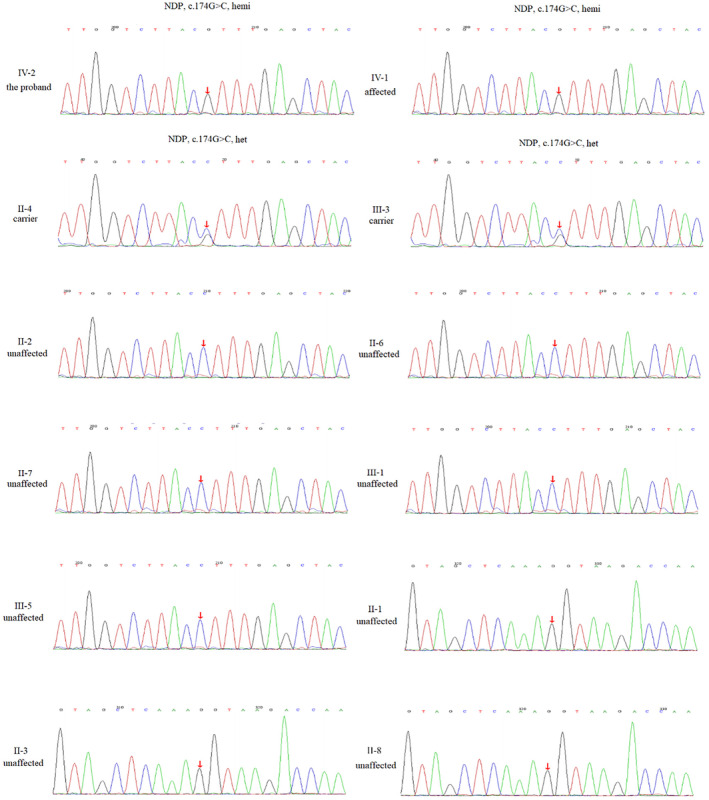
Detected mutations in the *NDP* genes in patients with Norrie disease. Partial sequences of *NDP* from the Norrie disease. Patients (IV‐2, IV‐1: Hemizygous for the c.174G>C genotype), controls (II‐1, II‐2, II‐3, II‐6, II‐7, II‐8), and carriers (II‐4, III‐3: Heterozygous for the genotype).

**FIGURE 4:**
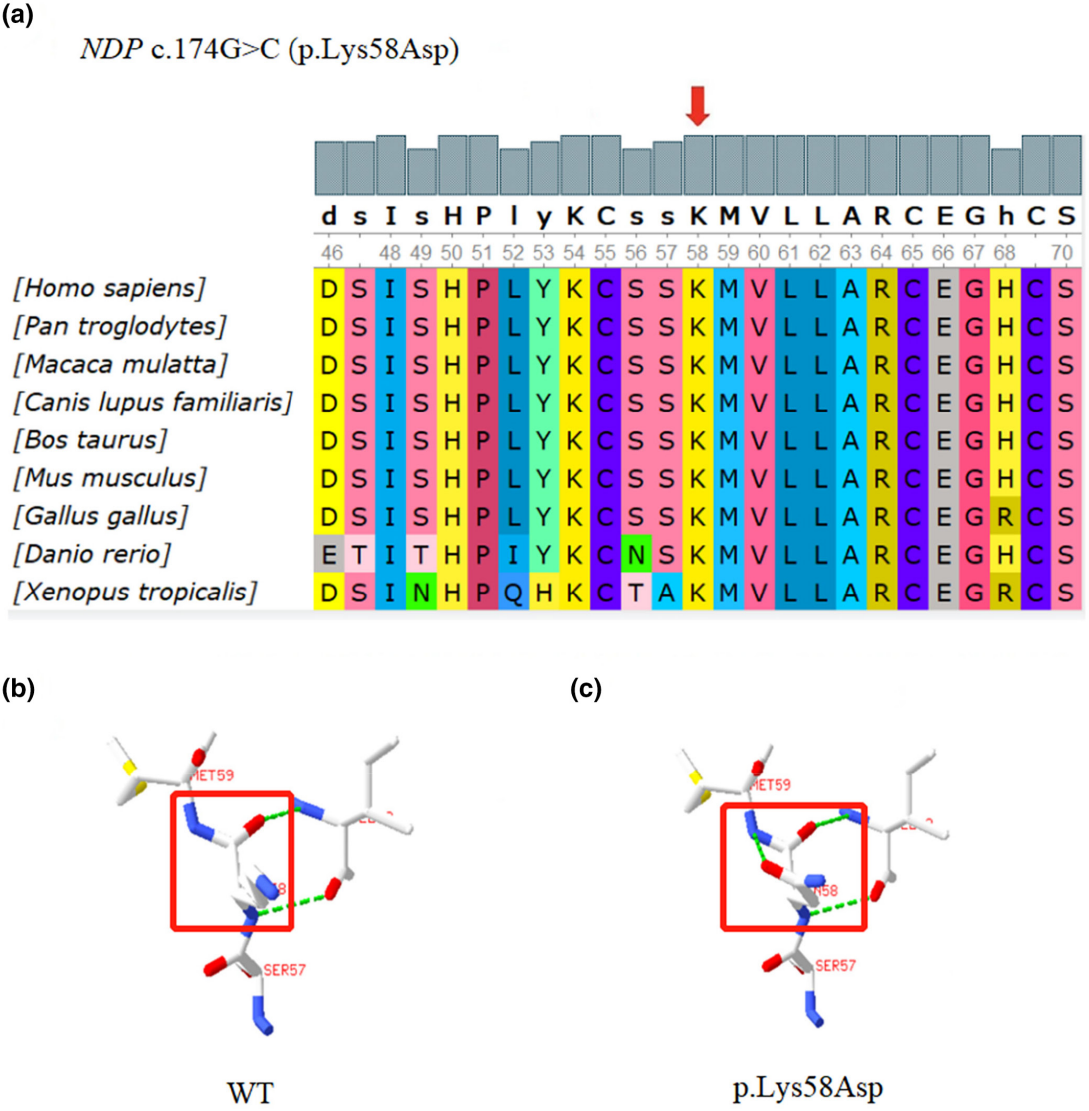
Phylogenetic conservation analysis of c.174G>C from different species and structural prediction of protein. (a) The red arrow represents the mutation site. Multiple sequence alignments revealed that c.174 in *NDP* gene were extremely evolutionarily conserved. (b, c) The simulation program SWISS‐MODEL was used to predict the influences of mutations in the Norrie protein. The results showed a great difference between the structure of the mutated and wild‐type, indicating a structure and functional change.

To elucidate the genetic factors underlying the retinal malformation in this family, next generation sequencing was performed. This analysis revealed a hemizygotic missense variant c.174G>C, in the *NDP* gene of the proband (Figure 3). Notably, this variant has not been previously reported in the database such as the 1000 genome (G1000), EXAC, ESP6500, dbSNP, and internal databases. To assess the potential impact of the variant on protein structure, four bioinformatics methods were employed, and all indicated its deleterious nature (Table 1). Furthermore, multiple orthologous sequence alignments (MSA) demonstrated a high conservation of the affected amino acid across different species (Figure 4a). Specifically, the variant resulted in the substitution of an alkaline amino acid (Lysine) with an acidic amino acid (Asparagine). Three‐dimensional modeling using SWISS‐MODEL revealed that the c.174G>C (p.Lys58Asp) alteration led to functional abnormalities and compromised the overall structural stability of the protein (Figure 4b,c).

**TABLE 1:** Summary of deleteriousness prediction methods analyzed mutation c.174G>C of *NDP* in our study.

Name	Category	Score	Deleterious threshold	Information used
SIFT	Function prediction	0.006	<0.05	Protein sequence conservation among homologs
PolyPhen−2	Function prediction	0.995	>0.5	Eight protein sequence features, three protein structure features
MutationTaster	Function prediction	1	>0.5	DNA sequence conservation, splice site prediction, mRNAstability prediction and protein feature annotations
GERP++	Function prediction	5.15	>0.5	Protein sequence conservation among homologs

After performing bioinformatics analysis, we proceeded with Sanger sequencing to confirm the presence of the identified variation in additional family members. The sequencing results revealed that the proband's brother IV‐1 carried the c.174G>C allele in a hemizygous state. Furthermore, the proband's mother (II‐4) and grandmother (III‐3) were identified as carriers of the mutation. Conversely, the unaffected family members, including II‐1, II‐2, II‐3, II‐6, and II‐7, II‐8, did not exhibit this variant. Pedigree analysis demonstrated the co‐segregated of the c.174G>C (p.Lys58Asp) variant with the disease phenotype within this family. According to the established standards and guidelines of the American College of Medical Genetics and Genomics (ACMG), the mutation of c.174G>C in *NDP* gene was predicted to be pathogenic.

## 4 DISCUSSION

Norrie disease (ND) is a rare X‐linked recessive retinal disorder characterized by severe ocular abnormalities, including retinal dysplasia with early vascular proliferation, retinal detachment, cataract, atrophic irides, corneal opacification, and eventually ocular shrinkage in affected males. In addition to the ocular manifestations, some affected patients may also experience varying degrees of mental retardation, cognitive decline, and sensorineural deafness as they age (Chen et al., 1992; Riveiro‐Alvarez et al., 2005; Rodríguez‐Muñoz et al., 2018). The pathogenic variations responsible for ND are found in the *NDP* gene, 205 variations according to the HGMD v 2022.1 database and 201 variations genetic alterations have been reported in this gene (Wawrzynski et al., 2022), chromosomal rearrangements, frame‐shift variants (Wang et al., 2022), nucleotide insertions (Andarva et al., 2018), splice site variations (Zhou et al., 2021), and mutations in the 5'UTR (Jia & Ma, 2021). The *NDP* gene, located on chromosome Xp11.4, encodes the secreted protein Norrin, which consists of 133 amino acids and contains the cystine‐knot domain (Meitinger et al., 1993).

However, the precise relationship between variations in the *NDP* gene and retinopathy remains to be fully elucidated. To date, *NDP* gene variants are associated with various retinopathy‐related diseases, including X‐linked familial exudative vitreoretinopathy (XL‐FEVR; OMIM #305390), Coat's disease (OMIM #300216), and retinopathy of prematurity (ROP), have similar ocular features to ND. Therefore, a thorough clinical examination should be conducted before genetic analysis in cases of retinopathy.

In this study, we present a family with a notable history of typical ND. The proband exhibited retinal detachment in both eyes, presenting as a “white eyeball” at the age of 2 months, and experienced progressive vision loss by the age of 8 months. Anterior segment photographs and B‐mode ultrasonography findings revealed bilateral retinal detachment and a lack of retinal nerve cells. XL‐FEVR, characterized by a slower progression and typically manifesting blindness in adolescence, was ruled out as a potential diagnosis (Sızmaz et al., 2015). Moreover, the proband's brother had been blind since childhood, while his uncle and great‐uncle had also experienced blindness since childhood (specific details are unknown) and died around the age of 10. In contrast no abnormalities were observed in any female members of the family. These findings strongly suspected diagnosis of ND, while autosomal conditions such as persistent fetal vasculature (PFV) related to the ATOH7 gene on chromosome 10q21.3 were excluded.

Exome sequencing was performed in the family, revealing a hemizygotic missense variant c.174G>C in the *NDP* gene of the proband. This variant was inherited from the mother (III‐4) and grandmother (II‐4) and was found to be associated with the disease states in individuals III‐4, IV‐1, and IV‐2. Considering the similar symptoms observed in individual II‐8, we suspected that individual I‐2 was also a carrier of the variant.

The variant was not found in the HGMD database, suggesting that it is a novel variant (PM2). Two missense changes occurring at the same position have been reported, this novel missense amino acid change is considered moderate evidence but cannot be assumed to be pathogenic (PM5) (Fuentes et al., 1993; Li et al., 2018). Bioinformatics tools predict it might be a pathogenic variant, further evaluation was conducted by Multiple orthologous sequence alignments (MSA) and SWISS‐MODEL. The MSA result demonstrated a high conservation of the affected amino acid across different species. While the SWISS‐MODEL revealed a structural change of the protein. This mutation occurs in exon 2 at codon 58, which is part of the cystine knot domain. It is worth noting that most *NDP* gene mutations associated with ND are related to this specific domain, such as P98L (c.293C>T) and S111X (c.332C>A) (Nikopoulos et al., 2010). Besides, missense mutations affecting cysteine residues are predicted to have a critical impact on the structure of the norrin homodimer as these residues are required for the formation of cysteine intermolecular disulfide bridges. These mutations universally cause “severe” retinal disease of bilateral mixed tractional and exudative retinal detachment, which is similar to the phenotype of the proband (PP4) (Riveiro‐Alvarez et al., 2005; Rodríguez‐Muñoz et al., 2018). These analyses consistently indicated a deleterious effect of the c.174G>C variant in the *NDP* gene (PP3). Subsequent Sanger sequencing also confirmed the co‐segregated of the variant with the disease phenotype in the family (PP1‐M). Consequently, the missense variant in exon 2 of *NDP* (NM_000266.4; c.174G>C, p. Lys58Asp) was classified as “likely pathogenic” according to the ACMG (PM2 + PM5 + PP1‐M + PP3 + PP4).

Currently, the treatment options for Norrie disease mainly included keratoplasty, lens dissection, early vitrectomy, and early retinal laser photocoagulation (Clevers, 2004; Walsh et al., 2010). In this particular case, we proposed retinal detachment repair, vitrectomy, cataract phacoemulsification, and aspiration after suprachoroidal cavity drainage for the proband. However, the guardian ultimately declined surgical treatment due to the poor prognosis. At present, the most effective prevention method of Norrie disease is prenatal genetic diagnosis (Wu et al., 2017). In this case of the proband's mother being carrier, we recommended prenatal genetic diagnosis for future pregnancies. Additionally, the occurrence of ND may also be observed in earlier gestational age fetuses. Therefore, in subsequent pregnancies of the proband's mother, it is important for the doctor to monitor fetal ocular changes through ultrasound in the second trimester. This approach will facilitate the early detection of abnormal fetuses and enable prenatal diagnosis.

In conclusion, we have described a novel mutation in the *NDP* gene in a Chinese family with Norrie disease. The clinical findings and bioinformatics analysis strongly suggest that the identified variant is disease‐associated variant. Our discovery expanded the mutation spectrum of ND and holds significant diagnostic implications for the patients with the same mutation. Further research is warranted to investigate the molecular mechanisms and clinical heterogeneity of Norrie disease in future studies.

## AUTHOR CONTRIBUTIONS

Each author's contributions list as follows. Keke Jiang and Shuying Wang: Supervision, Conceptualization Supervision, final approval of the version to be submitted. Huixin Sun: The conception and design of the study, acquisition of data, analysis, and interpretation of data, and drafting of the article. Chuanzhi Peng and Nan Li: Revising the article critically for important intellectual content. Shuchan Li: Data curation, Formal Analysis, Software. Jie zhang: Visualization. Rongyu Gao: Resources, Investigation.

## FUNDING INFORMATION

This research was funded by The Bai Qiu En · Langmu Young Ophthalmology Research Fund (BJ‐LM2019005J) and the general project of the natural science foundation of Shandong province (grant number ZR2021MH411).

## CONFLICT OF INTEREST STATEMENT

The authors declare no conflict of interests.

## ETHICS STATEMENT

This study was approved by the Ethics Committee of WeiFang Eye Hospital (approval number: 2022‐04‐01).

## Data Availability

The data that support the finding of this study are available from the corresponding author upon reasonable request.
